# Flimmernde Skotome nach COVID-Impfung: Eine weitere mögliche Impfnebenwirkung?

**DOI:** 10.1007/s00347-021-01435-z

**Published:** 2021-06-21

**Authors:** K. Gabka, S. Groselli, M. Ulbig

**Affiliations:** Klinik und Poliklinik für Augenheilkunde, Klinkum rechts der Isar, Technische Universität München, Ismaninger Str. 22, 81675 München, Deutschland

## Anamnese

Eine 20-jährige Patientin stellte sich erstmalig mit seit 2 Wochen persistierender beidseitiger Wahrnehmung von hellen flimmernden Flecken im zentralen Sehfeld in der Ambulanz der Augenklinik des Klinikum rechts der Isar in München vor. Die Patientin gab an, die beidseits hellen und teilweise flimmernden Flecken um den Punkt des zentralen Sehens plötzlich bemerkt zu haben und dass diese weiterhin unverändert bestünden. Eine Sehschärfenverschlechterung oder Metamorphopsien habe sie nicht bemerkt. Im Amsler-Gitter war eine grobe Einzeichnung der Flecken durch die Patientin möglich, diese zeigten sich beidseits parazentral temporal. Anamnestisch gab sie weiterhin an, dass die Beschwerden einen Tag nach erfolgter Corona-Impfung mit dem Impfstoff Vaxzevria® (AstraZeneca, Cambridge, UK) auftraten. Weitere Symptome wie Fieber, Schüttelfrost und Kopfschmerzen hielten für 2 Tage an. An chronischen Krankheiten würde die Patientin nicht leiden, auch nehme sie außer einer oralen Kontrazeption keine weiteren Medikamente dauerhaft ein.

## Klinischer Befund

**Figure 1:**
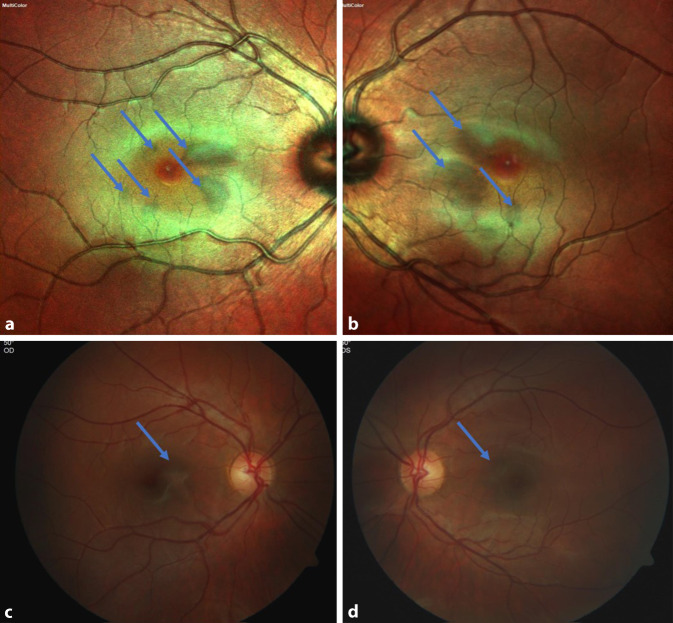

Der bestkorrigierte Visus lag am rechten Auge bei 1,0 p (+0,50 sph./−0,25 cyl./Achse 156°) und am linken Auge bei 1,0 (+0,25 sph./−0,50 cyl./Achse 176°). In der Spaltlampenuntersuchung zeigte sich ein regelrechter und reizfreier vorderer Augenabschnitt mit klaren optischen Medien beidseits. Fundoskopisch stellte sich die Papille beidseits randscharf begrenzt und vital gefärbt sowie die Makula beidseits mit dezenten Aufhellungen nahe der Fovea dar (Abb. 1).

**Figure 2:**
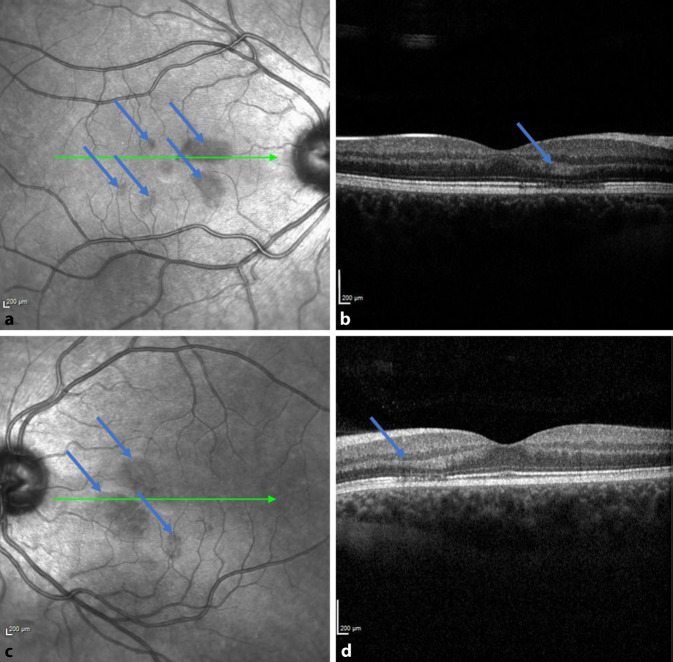

**Figure 3:**
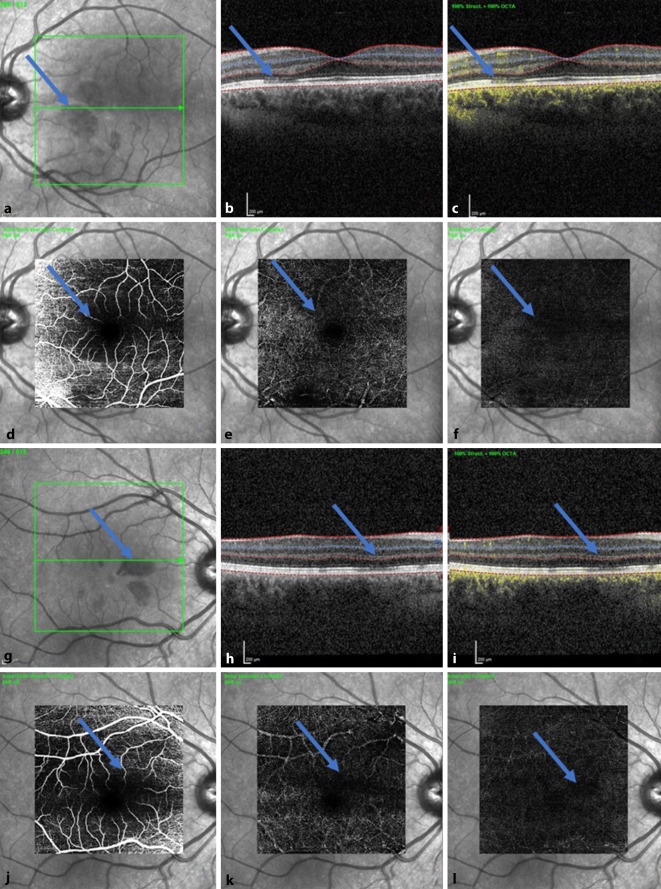

In der optischen Kohärenztomographie (Spectral-Domain-optische Kohärenztomographie, SD-OCT, Heidelberg Instruments) zeigten sich in der Übersichtsaufnahme/Infrarotaufnahme der Makula dunkle ovale bzw. petaloide perifoveale Läsionen mit der Spitze zur Fovea zeigend (Abb. 2a, b). Diese entsprachen anatomisch den Skotomen, die die Patientin im Amsler-Gitter eingezeichnet hatte. Die SD-OCT-Schnittbilder durch die Läsionen zeigten hyperreflektive Plaques in der äußeren plexiformen Zone, die zu einer fokalen Unterbrechung der inneren und äußeren Segmente („IS/OS junction“) und ellipsoiden Zone führten (Abb. 2c, d). In der SD-OCT-Angiographie-Kohärenztomographie (Spectral-Domain-optische Kohärenztomographie [SD-OCT-A]) stellten sich v. a. am rechten Auge reduzierte Flusssignale im tiefen retinalen Kapillarplexus dar (Abb. 3).

## Wie lautet Ihre Diagnose?

## Diskussion

Die akute makuläre Neuroretinopathie (AMNR) ist eine seltene Erkrankung, die in der Regel gesunde Frauen im Alter bis zum 30. Lebensjahr betrifft. Die Patientinnen berichten über ein plötzliches Auftreten von parazentralen Skotomen teilweise mit Visusminderung oder verschwommenem Sehen. In bis zu 54,4 % der Fälle der AMNR wird von einem bilateralen Auftreten berichtet [1].

Die mit AMNR assoziierten typischen Läsionen sind klinisch kaum sichtbar und werden gruppiert um die Fovea als flache keil- oder blütenblattförmige Läsionen von rötlich-brauner oder oranger Farbe oder selten als hypopigmentiert oder gräulich-weiß beschrieben [1–3]. Zur Diagnosesicherung sind weiterführende Untersuchungen wie das OCT, OCT‑A, die Fundusautofluoreszenz und die Infrarotbildgebung ausschlaggebend, die Fluoreszenzangiographie sowie elektrophysiologische Tests (ERG) zeigen sich meist unauffällig [1, 3, 4].

Die genaue Ursache der AMNR ist noch nicht geklärt. Möglicherweise wird die AMNR durch eine mikrovaskuläre Ischämie der Choriokapillaris verursacht. Es wird angenommen, dass diese Gefäßschicht aufgrund ihrer geringen Autoregulation und ihres hohen Anteils von α‑adrenergen Rezeptoren anfällig für transitorische ischämische Insulte ist. Verursacht wird diese Ischämie möglicherweise durch die Freisetzung von Katecholaminen, den Gebrauch von Sympathomimetika, eine Hyperviskosität durch Leukozytose, eine erhöhte Kapillarpermeabilität, eine Koagulopathie, eine Thrombozytendestruktion oder eine konsumtive Koagulopathie [1, 5, 6]. Am häufigsten ist die Krankheit mit einer vorangegangenen unspezifischen grippeähnlichen Erkrankung oder Fieber (47,5 %) und mit der Einnahme von hormonellen Kontrazeptiva (35,6 %), wie auch bei unserer Patientin, assoziiert. In selteneren Fällen ist von einer Assoziation mit starkem Kaffeekonsum, der Exposition gegenüber Epinephrin/Ephedrin (7,9 %) oder einem vorhergehenden Trauma (5,9 %) berichtet worden. Weiterhin sind Fälle mit systemischem Schock (5,0 %), schwerer arterieller Hypotonie und sehr starkem Koffeinkonsum beschrieben [1, 2, 7, 8]. In unserem Fall ist der Erkrankung eine Impfung mit Vaxzevria® von AstraZeneca vorausgegangen. Virgo und Mohamed beschrieben bereits im Juli 2020 eine mögliche Assoziation zwischen AMNR und der SARS-CoV-2-Infektion [9]. Von einer impfinduzierten AMNR liegt jedoch derzeit noch kein Bericht vor. Da grippeähnliche Prodromi bei AMNR typisch sind, ist zu vermuten, dass die AMNR durch die COVID-Impfung ausgelöst worden sein könnte. Ähnlich wie bei einem Virusinfekt könnte die Impfung eine Immunreaktion ausgelöst haben.

**Diagnose:** Akute makuläre Neuroretinopathie (AMNR): Zufall oder Folge der COVID-Impfung mit dem AstraZeneca-Impfstoff?

Eine spezifische Therapie für die AMNR gibt es nicht, von einer spontanen Besserung berichten 48 % der Fälle [3]. Tatsächlich konnte mittels OCT-Scans eine Erholung der ellipsoiden Zone bzw. eine partielle Wiederherstellung der inneren und äußeren Photorezeptorsegmente (IS/OS) mit dem Verschwinden der rötlich-braunen Läsionen bzw. hyperreflektiven Plaques festgestellt werden. Eine Ausdünnung der äußeren Körnerschicht scheint jedoch trotz Behebens der IS/OS-Defekte bestehen zu bleiben [4, 10, 11].

## Fazit für die Praxis

Von einer möglichen impfinduzierten AMNR liegen derzeit keine weiteren Fallberichte vor. Die AMNR ist eine sehr seltene Erkrankung am Auge, die nicht immer korrekt diagnostiziert wird. Typisch für die AMNR sind die grippeähnlichen Symptome vor Ausbruch, wie sie im Rahmen der Impfung bei der Patientin beobachtet wurden. Die beschriebenen thromboembolischen Effekte des Vaxzevria®-Impfstoffs von AstraZeneca könnten möglicherweise auch retinal auftreten und dort eine vaskuläre Ischämie im tiefen Netzhautplexus bedingen, wie sie auch bei AMNR angenommen wird. Ob es sich tatsächlich um eine Nebenwirkung der COVID-Impfung bzw. speziell des Vaxzevria®-Impfstoffs von AstraZeneca bei jungen Frauen handelt, bleibt weiterhin unklar. Da die AMNR eine sehr seltene und meist selbstlimitierende Erkrankung ist, sollte auf die Impfung keinesfalls verzichtet werden.
